# A comparison between lesions found during meat inspection of finishing pigs raised under organic/free-range conditions and conventional, indoor conditions

**DOI:** 10.1186/2055-5660-1-4

**Published:** 2015-04-16

**Authors:** Lis Alban, Jesper Valentin Petersen, Marie Erika Busch

**Affiliations:** Danish Agriculture & Food Council, Axeltorv 3, Copenhagen V, DK-1609 Denmark

**Keywords:** Pig health, Production system, Meat inspection, Animal welfare

## Abstract

It is often argued that pigs raised under less intensive production conditions – such as organic or free-range – have a higher level of animal welfare compared with conventionally raised pigs. To look into this, an analysis of data from a large Danish abattoir slaughtering organic, free-range, and conventionally raised finishing pigs was undertaken. First, the requirements for each of the three types of production systems were investigated. Next, meat inspection data from a period of 1 year were collected. These covered 201,160 organic/free-range pigs and 1,173,213 conventionally raised pigs. The prevalence of each individual type of lesion was calculated, followed by a statistical comparison between the prevalences in organic/free-range and conventional pigs. Because of the large number of data, the P-value for significance was lowered to P = 0.001, and only biological associations reflecting Odds Ratios above 1.2 or below 0.8 were considered to be of significance.

The majority of the lesion types were recorded infrequently (<4%). Only chronic pleuritis was a common finding. A total of 13 lesion types were more frequent among organic/free-range pigs than among conventional pigs - among others old fractures, tail lesions and osteomyelitis. Four lesion types were equally frequent in the two groups: chronic pneumonia, chronic pleuritis, fresh fracture, and abscess in head/ear. Four lesion types were recorded less frequently among organic/free-range pigs compared with conventionally raised pigs. These included abscess in leg/toe, hernia and scar/hock lesion.

Possible associations between the individual lesion types and the production systems - including the requirements for each system - are discussed. The results emphasize the importance of using direct animal based parameters when evaluating animal welfare in different types of production systems. Moreover, individual solutions to the health problems observed in a herd should be found, e.g. in collaboration with the veterinary practitioner and other advisors.

## Background

Animal welfare in pig production is discussed continuously in Denmark and elsewhere. It is argued by some parties that pigs raised under less intensive production conditions - such as organic or free-range – have a higher level of animal welfare compared with pigs raised under conventional, indoor conditions.

In early 2014, the Danish discussion was taken to the political level, and stakeholders were invited to contribute to the discussion of how to improve animal welfare in the pig production. One of the questions to address was whether animal welfare was, objectively seen, higher in organic/free-range production compared with conventional production.

### About Danish pig production

In Denmark, pig production plays a substantial role, representing an export value of €4.3 billion out of a total of €81.6 billion [1].

The largest Danish abattoir company is Danish Crown. Almost all pigs raised under special animal welfare contracts in Denmark are slaughtered in one specific plant owned by Danish Crown. The company “Friland A/S”,”, owned by Danish Crown, has special contracts with farmers, who raise pigs according to the rules set for organic pig production (Friland økologiske svin) or the rules set for “Frilandsgrisen” which deals with non-organic outdoor production. The pig genetics are similar in these two types of production systems as well as in the conventional production, and almost all male pigs are castrated.

In the following, the requirements for the different types of production systems are described in brief.

### Description of the production systems included in the study

#### Requirements for ”Frilands økologiske svin” (organic production)

Organic pig production in Denmark must comply with EU rules for organic production [2]. In addition, the company “Friland A/S” has – in collaboration with the Danish Animal Welfare Society – developed a set of requirements to ensure a high level of animal welfare [3]. Friland’s organic pigs are born in huts on a field and stay with the sow until they are at least 7 weeks old. Each sow with piglets has an area of minimum 300 m^2^. Tail-docking is prohibited. After weaning, the piglets can be moved to indoor pens with plenty of bedding to facilitate rooting and resting. Permanent access to an outdoor area must be provided. The rules for the use of antimicrobials are stricter than for the other production systems; if a pig goes through two treatments courses with antimicrobials, the pig is no longer considered organic. Roughage must be fed to all pigs. This constitutes substrate for the pigs’ natural rooting behavior and may also benefit gastro-intestinal health. During summer, the pigs must have access to cooling, either by the use of a shower or a mud bath. The most relevant of these rules are described in Table 1. On top of the ordinary price paid for the pigs by the slaughterhouse company, the farmer also receives a premium for organic pigs [4]. In the first quarter of 2014, the premium for an average organic carcass with a weight of 84 kg was €113.7 (J.P. Nannerup, Head of Department, Friland A/S, personal communication, June 2014). The premium is not paid if any of the following lesions are found during meat inspection: tail lesion, healed rib fracture, chronic infectious arthritis, old fracture, osteomyelitis, skin corrosion, chronic peritonitis, hernia, pyemia, scar/hock lesion as well as abscess in the leg, toe, head, ear, middle or hind part.

**Table 1:** **Description of requirements to three different pig production systems commonly used in Denmark**

	Friland Organic	Friland Free-range	Conventional
Source of information	[3],	[3]	[5]
	[35],		
	[36]		[37, 38]
Born outdoors in huts	Required	Required	Optional
Age at weaning (minimum)	7 weeks	5 weeks	(3^a^) 4 weeks
Tail docking	Prohibited, exemption can be granted	Prohibited, exemption can be granted	Allowed, if needed
Teeth reduction	Teeth cutting prohibited, grinding allowed within the first 3 days of pigs’ life if needed	Teeth cutting prohibited, grinding allowed within the first 3 days of pigs’ life if needed	Teeth cutting prohibited, grinding allowed within the first 3 days of pigs’ life if needed
Resting area with bedding (weaners/growers/ finishers)	Required – Minimum size of area defined for each weight group	Required – Minimum size of area defined for each weight group	Bedding optional - Straw or other material for rooting is required
Floors (weaners/growers/ finishers)	At least 50% of the required minimum area should have solid floor (outdoors this can be replaced by drained^b^ floor)	Solid floor in the resting area, at least 50% solid floor or deep straw in outdoor area (exemption can be granted)	At least 33-50% solid or drained floor (depending on age)^c^
Access to outdoor facility	Required	Required	Optional
Pen area required per finishing pig (at 100 kg)	2.3 m^2^ (1.3 m^2^ indoors, and 1.0 m^2^ outdoors)	1.2 m^2^ (min. 0.50 m^2^ outdoors)	0.65 m^2^
Access to roughage for weaners/growers/finishers	Required	Optional	Optional
Veterinary advisory service contract	Required	Required	Optional for small herds and required for large herds
Initiate treatment with antimicrobials	Not allowed^d^	Allowed (all herds are covered by veterinary advisory service contracts)	Allowed if the herd is covered by a veterinary advisory service contract
Withdrawal time related to use of antimicrobials	Twice as long as described in the regulation	Twice as long as described in the regulation	As described in the regulation

^a^Piglets can be weaned at an age of 3 weeks, if they are transferred to nursery rooms with controlled climate.

^b^Drained floor defined as maximum 10% openings.

^c^Ban on fully-slatted floors applies from July 2000 for new buildings and for all housing by July 2015.

^d^A pig that has been through two courses of treatment with antimicrobials is no longer considered organic.

#### Requirements for ”Frilandsgrisen” (free-range)

Similarly to the organic pig production, producers of “Frilandsgrisen” (free-range) must comply with the rules developed by the company “Friland A/S” and the Danish Animal Welfare Society [3]. The most relevant of these rules are described in Table 1. Friland pigs are born in a hut on a field where they stay with the sow until they are 5 weeks of age. Tail-docking is prohibited. After weaning, the piglets can be moved to indoor pens with a resting area with bedding, as long as they have access to an outdoor area. The rules for the use of antimicrobials are stricter than for conventional production (Table 1). When the pigs are heavier than 20 kg they must have access to cooling by the use of a shower or a mud bath, if the average daily temperature is above 15Â°C. A premium is paid on top of the ordinary price for the pig meat [4]. In the first quarter of 2014, the premium for an average free-range carcass with a weight of 84 kg was €24.5 (J.P. Nannerup, Head of Department, Friland A/S, personal communication, June 2014). As for organic pigs, the premium is not paid if any of the lesions described in section 1.2.1 are found during meat inspection – the only difference is that tail lesions are accepted for free-range pigs.

#### Requirements for finishing pigs from conventional production

All farms, delivering pigs to Danish Crown’s abattoirs have to comply with a set of specified requirements for the production of conventional pigs. The requirements are described in a private standard called the Danish Product Standard [5]. This standard is equivalent to the German Qualität und Sicherheit (QS) and the Dutch IKB, and hence fulfills international requirements to pig-raising, traceability, and use of antimicrobials. Tail docking is not prohibited in the conventional production as long as the requirements of the Danish legislation on protection of pigs are fulfilled. The farmer is allowed to initiate treatment of diseased animals if there is a veterinary advisory service contract in place. Such contracts are always required for large herds (herds with >300 sows, >3,000 finishers or >6,000 piglets). The required minimum pen area per pig weighing 100 kg is 0.65 m^2^ (Table 1). In pens with pigs that are heavier than 20 kg, a shower or equivalent device must be installed and used to control the body temperature of the pigs when necessary.

### Using meat inspection data for measuring animal welfare

In Denmark, finishing pigs are subjected to ante mortem and post mortem inspection at the abattoir performed by a specially trained inspector – such as a veterinarian or a technician. Hence, every slaughter animal is inspected according to the current meat inspection circular [6].

One common coding system with more than 70 codes is in use in all Danish abattoirs [6]. Observed lesions are recorded routinely by use of a finger-touch terminal placed adjacent to the meat inspector. Data are transferred to a national database owned by the slaughterhouses and the veterinary authorities.

Some variation exists with respect to how the coding system is used – primarily between plants and secondarily between meat inspectors within a plant. It is attempted to keep these sources of variation low by use of inter-calibration courses arranged at regular intervals by the Danish Veterinary and Food Administration for the meat inspectors. However, on some plants, the threshold is low when it is decided if a lesion is of such an extent that it should be recorded, whereas on other plants the threshold is higher. The between-plant variation was eliminated from the present study because only one plant was included. On this plant, organic (Frilands økologiske svin), free-range pigs (Frilandsgrisen) and conventionally raised pigs (Danish Product Standard) are slaughtered. On the plant all types of pigs are inspected by the same meat inspection staff, using the same coding system and complying with the same meat inspection circular. A total of 22 technicians and five veterinarians are involved in the meat inspection, working either part or full time. The type of meat inspection performed at the time of the study is called supply chain meat inspection and involved palpation of the lungs and their associated lymph nodes as well as visual inspection of the intestines and associated lymph nodes. The heart was not opened routinely, and the mandibular lymph nodes were not incised routinely. In case of suspicion, the pig (carcass, plucks and intestines) was subjected to traditional - or even extended - meat inspection at the rework area [7].

We decided to look into data from meat inspection. Such data can be seen as one group out of several indicators of animal welfare. We did not go into the discussion of scoring the negative impact of each of the individual types of lesions on the welfare of the animal. However, it should be noted that most of them reflect chronic conditions, implying that they have been long-lasting.

### Aims

1. To compare the health, at the time of slaughter, of finishing pigs raised in organic or free-range production systems with the health of finishing pigs raised under conventional conditions. Meat inspection findings were to be used as an indicator for health.
2. To investigate the possible associations between the individual lesion types and the production systems – with a specific focus on the requirements for each system.

## Results and discussion

### Statistical evaluation

An initial comparison of the prevalence of the total of all lesions did not reveal any differences between organic/free-range (39.4%) and conventional production (39.1%). However, when chronic pleuritis was subtracted from the total number of lesions, a difference was observed; the prevalence of lesions in organic/free-range production was 20.3% compared with 15.2% in the conventional production (OR = 1.3, P < 0.0001) (Table 2).

**Table 2:** **Prevalence of total number of lesions – as well as all lesions minus chronic pleuritis - recorded during meat inspection in organic/free-range finishing pigs (N = 201,160) compared with conventionally raised finishing pigs (N = 1,173,213), for one Danish slaughterhouse, covering 1 year from October 1, 2012, to September 26, 2013**

	Total No. of lesions				Total No. of lesions minus chronic pleuritis			
Production system	No. of lesions	Prevalence (%)	No. of slaughtered pigs	Odds Ratio	No. of lesions	Prevalence (%)	No. of slaughtered pigs	Odds Ratio*
Organic/free-range	79,174	39.4	201,160	1.01	40,830	20.3	201,160	1.34
Conventional	459,239	39.1	1,173,213	1.00	178,428	15.2	1,173,213	1.00

*P-value to test the statistical difference between the observed prevalences was <0.0001.

The majority of the lesion types were recorded infrequently (<4%). Only chronic pleuritis was a common finding with a prevalence of 19% among organic/free-range pigs and 24% among conventional pigs. A total of 13 lesion types were more frequent among organic/free-range pigs compared with conventional pigs. These covered, among others, old fracture, tail lesion/tail infection and osteomyelitis. Four lesion types were equally common in the two groups: chronic pneumonia, chronic pleuritis, fresh fracture, and abscess in head/ear. Four lesion types were recorded less frequently among organic/free-range pigs compared with conventionally raised pigs. These included abscess in leg/toe, hernia and scar/hock lesion (Table 3).

**Table 3:** **Prevalence of lesions recorded during meat inspection in organic/free-range finishing pigs (N = 201,160) compared with conventionally raised finishing pigs (N = 1,173,213), for one Danish slaughterhouse, covering 1 year from October 1, 2012, to September 26, 2013, sorted by Odds Ratio**

Lesion type	Prevalence (%)		Odds Ratio	P-value for comparison
	Organic/free-range	Conventional		
HIGHER RISK*				
Healed rib fracture	0.73	0.19	3.8	<0.0001
Eczema/insect bite	2.41	0.73	3.4	<0.0001
Tail lesion – local	2.37	0.76	3.2	<0.0001
Chronic infectious arthritis	0.87	0.27	3.2	<0.0001
Milk spotted liver	2.60	0.90	3.0	<0.0001
Old fracture	0.20	0.09	2.2	<0.0001
Osteomyelitis	0.34	0.16	2.1	<0.0001
Tail lesion/tail infection	0.18	0.09	2.0	<0.0001
Skin corrosion	0.10	0.06	1.8	<0.0001
Abscess in mid-part	0.47	0.30	1.6	<0.0001
Abscess in hind part	1.30	0.82	1.6	<0.0001
Chronic peritonitis	1.11	0.74	1.5	<0.0001
Chronic pericarditis	1.67	1.32	1.3	<0.0001
EQUAL RISK*				
Chronic pneumonia	0.35	0.30	1.2	0.0006
Abscess in head and ear	1.81	1.90	0.9	0.0039
Fresh fracture	0.13	0.17	0.8	<0.0001
Chronic pleuritis	19.06	23.94	0.8	<0.0001
LOWER RISK*				
Abscess in leg/toe	0.74	1.03	0.7	<0.0001
Hernia	0.93	1.36	0.7	<0.0001
Pyemia	0.01	0.02	0.5	0.0007
Scar/hock lesion	1.47	3.41	0.4	<0.0001

*Biological significance assessed at OR > 1.2 or OR < 0.8. Statistical significance only assessed at P < 0.001.

### Evaluation of findings

This is the first time that a large dataset has been investigated allowing a deeper comparison between the various lesions found during meat inspection of organic/free-range finishing pigs and conventionally raised finishing pigs. The analysis shows a significantly higher prevalence of a long list of lesions (n = 13) in organic/free-range pigs compared with conventionally raised finishing pigs. However, for some lesions, the prevalence was the same (n = 4) or even lower in organic/free-range production (n = 4). Since the pigs in the two types of production systems are genetically similar, there is no reason to believe that the differences are caused by genetic differences.

In the following, the possible mechanisms leading to the observed associations between the individual lesions and the type of production system are discussed. Moreover, comparisons with results of related studies were made. The main paper to compare with was made by Bonde et al. [8] who in 2005 undertook a study on meat inspection data from 16 organic and 52 conventional Danish pig herds. In that study, lesions were grouped according to organ system, probably as a way to increase the number of findings to compensate for the small size of the data set. Therefore, comparisons between the two studies were only made where it was possible. In line, we compared our results with a Swedish study undertaken by Heldmer et al. [9], which included around 110.000 organic pigs and 27 million conventional pigs. The Heldmer et al. study only reported findings of arthritis, milk spots and pneumonia/pleuritis.

We suggest that the higher occurrence of a number of lesions observed during meat inspection of organic/free-range pigs can be linked to the following factors: 1. A higher level of tail biting, 2. Limited all-in all-out management (batch management) and poorer hygiene, 3. A humid/wet floor in parts of the pen, and 4. Squeezing by the sow during the suckling period. Furthermore, it is possible that there is a reluctance to use antimicrobials in organic and free-range production and that this will influence the occurrence of most of the lesion types caused by bacterial infections in these production systems. Please, see the following paragraphs for how we reached these hypotheses.

#### Lesions with a higher risk in organic/free-range pigs

Tail lesions were more frequent among organic/free-range pigs compared with conventional finishing pigs. This applied for both types of tail lesions recorded, i.e. tail injury that had led only to a local, well-demarcated lesion resulting in local condemnation (OR = 3.2), and tail lesions that were found to have caused a more widespread infection, thus being a more severe condition (OR = 2.0). The higher prevalence of tail lesions recorded in organic/free-range production might be related to the pigs having intact tails in these production systems, whereas the majority of the pigs from conventional production are tail-docked. In a review on tail-docking, Sutherland & Tucker [10] concluded that docking reduced the probability of tail biting behavior in pigs. It cannot be ruled out that intact tails and docked tails are not assessed in completely the same way by the meat inspection, simply because the tails have a different shape and size, and that this could be part of the reason for the higher prevalence of local tail lesions in organic/free-range pigs. However, the fact that the prevalence of tail lesions with more widespread infection was also higher in organic/free-range pigs suggests that there is a true difference between the two types of production systems. Tail lesions that involve local spread only will result in local condemnation, whereas tail lesions with further spread will result in total condemnation or approval for deboning only [6].

The finding that the risk of tail lesions is higher in organic/free-range production compared with conventional production is controversial, because the current belief is that tail-biting is related to a poor environment e.g. represented by slatted floor with no bedding and little opportunity for performing exploratory behavior [11]. Because of the difference in tail docking practices in the two types of production systems, our data cannot be used for a direct comparison of the pigs’ tendency to perform tail biting. However, data do suggest that the conditions in organic/free-range herds do not prevent tail biting and that the presumed positive effect of the environment in these herds does not outweigh the increased risk of tail biting in undocked pigs compared with docked pigs.

Osteomyelitis was also more frequent in organic/free-range pigs than in conventional pigs (OR = 2.1). Since tail lesions are a significant cause of osteomyelitis, it is likely that the higher prevalence of osteomyelitis in organic/free-range pigs is, at least partly, related to the higher occurrence of tail lesions [12, 13]. The prevalence of pyemia was apparently lower in organic/free-range pigs (OR = 0.5). However, the total number of cases was very low (organic/free-range: 24 out of 201,160 pigs versus 270 out of 1,173,213 conventional pigs). The phenomenon was probably caused by a habit of using the code osteomyelitis more frequently than pyemia at the abattoir, because the condition behind both lesions is often the same: an infected tail lesion leads to pyemic processes, some of which are presented as osteomyelitis. Osteomyelitis will result in total condemnation or approval for deboning only [6].

It might be questioned whether it is necessary to dock the tails of all piglets to prevent the development of tail lesions in a proportion of the pigs later in life. Here it should be considered that many farmers and their veterinarians might be inclined to tail-dock also to prevent the often severe sequels of tail lesions, such as septicemia, osteomyelitis, abscesses and increased mortality (including euthanasia).

In conventional production, the farmer usually has access to antimicrobials and can therefore easily initiate treatment of a pig with a tail lesion in accordance with the advice given by the veterinary practitioner. Similarly, in free-range production the farmer is allowed to initiate treatment of diseased pigs, because a veterinary advisory service contract is always required (Table 1). In organic pig production, by contrast, farmers are not allowed to initiate antimicrobial treatment. In addition, an organic pig that has been through two courses of treatment with antimicrobials is no longer considered organic (Table 1), and the producer will not receive the premium of €114. These rules might result in under-treatment, because the gross margin for pig production is low, which does not favor many extra visits by the veterinary practitioner – as also mentioned by Struve [14]. It is likely that under-treatment (or late treatment) of tail bitten pigs could result in a higher risk of the above mentioned sequels of tail lesions. Here, it should be mentioned that the antimicrobial consumption in organic pig production in Denmark is only 10-23% of the consumption seen in conventional production [15]. In line, Hegelund et al. [16] found that the consumption of antimicrobials was three times higher in conventional finishing pig herds compared with organic herds. Moreover, Hegelund et al. [16] found that 44% of the 16 organic producers included in their study did not use antimicrobials at all during the 1-year study period versus 15% of the conventional producers. The same pattern was found in the so-called Qualisafe Project conducted in 2007: 95% of 46 conventional herds as well as 93% of 27 free-range herds had reported use of antimicrobials whereas this was the case in only half of the 51 organic herds included in the study [17].

The withdrawal time after antimicrobial treatment is twice as long as required by the authorities in both organic and free-range production. The rule of doubling the withdrawal period should in principle not influence the choice on whether to treat a diseased animal or not because several antimicrobials have short withdrawal periods, e.g. penicillin procaine - which can be used for treatment of tail lesions – officially has a withdrawal period of 4 days in Denmark.

Abscess in the mid-part (OR = 1.6) and the hind part (OR = 1.6) can also, in some cases, be a result of a spreading of bacteria from a tail lesion. However, since these abscesses often occur as single abscesses without any signs of a concurrent pyemic condition, they probably can be caused by a number of other factors too, e.g. conditions in the pen environment. According to Bonde et al. [8] skin lesions and abscesses seen as one group were slightly less frequently recorded in organic pigs (4.8%) than in conventional pigs (5.0%). However, because of the grouping of lesions in the study by Bonde et al. [8], it is not possible to make a direct comparison between the two studies. Abscesses usually result in local condemnation [6].

Eczema/insect bite (OR = 3.4) were recorded more frequently in organic/free-range pigs compared with conventionally raised finishing pigs. These lesions are related to the outdoor environment and the open buildings – including eczema which may be caused by sun burning.

Milk spotted liver (OR = 3.0) was also recorded more frequently in organic/free-range pigs compared with conventionally raised finishing pigs. This is in accordance with Bonde et al. [8] and Heldmer et al. [9] who also found a significantly higher prevalence of milk spots at the time of slaughter among organic finishing pigs compared with conventionally raised pigs. The milk spots are due to exposure of the pigs to *Ascaris suum* which is often present to a high degree in pigs in an outdoor environment [18, 19]. Thus organic/free-range pigs will often be subjected to a higher level of infection with *Ascaris suum* before weaning than conventional pigs. In addition, they are probably also often subjected to a higher level of infection after weaning because of poorer hygiene in the pens and the connected outdoor area and more humid/wet floors. In organic/free-range herds, the level of cleaning and disinfection as well as the degree of all-in all-out management is usually considerably lower than in conventional herds. Furthermore, the use of solid floors will often lead to soiled areas of the floors.

The assumption that the floors can be more humid in organic/free-range herds is supported by the finding that skin corrosion was also recorded more frequently in organic/free-range pigs than in conventional pigs (OR = 1.8). Skin corrosion is probably a result of pigs resting in soiled areas of the pen. In organic/free-range production, solid floors are used to a much higher extent than in conventional production due to the specific floor requirements and the requirement for bedding (Table 1). However, slatted floors are better at draining urine and feces than solid floors.

The higher prevalence of chronic arthritis (OR = 3.2) recorded among organic/free-range finishing pigs compared with conventional finishing pigs is in accordance with findings in Swedish slaughter house data [9, 20]. The higher occurrence in organic/free-range pigs could have several causes. Firstly, it could be linked to a higher risk of infection with *Erysipelothrix rhusiopathiae* which can be found in the soil [21]. Secondly, a more humid pen environment and a poorer hygiene in the organic/free-range systems might result in a higher general infection pressure, including a higher pressure from pathogens causing arthritis (primarily *Mycoplasma hyosynoviae*). Thirdly, it is possible that joint injury caused by slippery floors could predispose the joint to arthritis. The risk of slippery floors is probably higher in organic/free-range systems because of the more widespread use of solid floors. A fourth explanation could be that the phenomenon was a result of impaired possibilities of diagnosing and subsequently treating infectious arthritis in the early stages of the disease when pigs are raised in more extensive production systems. Lastly, as speculated by Kugelberg et al. [21], organic/free-range pigs might have a higher prevalence of osteochondrosis because the development of osteochondrosis is promoted by mechanical stress in the joints and, thus, perhaps by exercise. However, even though osteochondrosis is associated with mild inflammatory reaction in the joints, this type of mild arthritis is probably not very often noticed at the meat inspection. Bonde et al.Â´s work [8] cannot be used for comparison because abscesses in legs, toes and chronic arthritis were summarized into one figure, which showed a slightly lower prevalence among organic pigs (2.7%) compared with conventional pigs (3.0%). This difference was statistically non-significant. Chronic arthritis usually leads to local condemnation [6].

Chronic pericarditis was more frequently recorded in organic/free-range production (OR = 1.3) compared with conventional production. The finding of a higher risk among organic/free-range pigs is in accordance with Bonde et al. [8] who found that there was a higher prevalence of heart- and circulatory lesions among organic finishers (2.5%) compared with conventional finishers (2.3%), though the difference was not statistically significant. Pericarditis was the principal lesion seen in this group of lesions. In a study of more than 1 million finishing pigs, Christensen [22] found that chronic pericarditis was associated with chronic pleuritis, chronic pneumonia, chronic peritonitis and chronic arthritis in the individual animal. Hence, the lesion is indicative of infectious disease. *Mycoplasma hyopneumoniae* probably often plays an important role in the development of pericarditis [23]. This is supported by the fact that chronic pericarditis is less prevalent in finishing pigs from Danish Specific-Pathogen-Free (SPF) herds than in pigs from SPF-herds that have been re-infected with *M. hyopneumoniae* (OR = 0.7) [22]. However, other agents, e.g. *Actinobacillus pleuropneumoniae*, *Streptococcus suis*, *Pasteurella multocida* and *Haemophilus parasuis*, can also cause pericarditis, either alone or as secondary infections [23–26]. There is no obvious explanation for the higher occurrence of pericarditis in organic/free-range pigs. However, it is possible that poorer hygiene and limited or lacking all-in all-out management also affect the occurrence of infections causing pericarditis. The lower consumption of antimicrobials in these herds might also play a role. Findings of chronic pericarditis will in uncomplicated cases lead to local condemnation [6].

The prevalence of chronic peritonitis recorded among organic/free-range finishing pigs was also higher than in conventional finishing pigs (OR = 1.5). Very little is known about the infectious agents causing peritonitis in pigs, but since peritonitis is associated with pericarditis on the individual animal level and on the batch level, the two lesions are probably often parts of the same disease complex [22, 27]. Chronic peritonitis usually leads to local condemnation [6].

There was a higher prevalence of healed rib fracture (OR = 3.8) and old fracture (OR = 2.2) among organic/free-range pigs compared with conventional pigs. This is most likely a result of injuries prior to weaning – where the sow by accident squeezes a piglet. This is a common problem in pig production, and in most conventional herds, it is alleviated by the use of farrowing crates. In the huts used in organic and free-range production, the risk of piglets being squeezed is higher and therefore, there is a focus on further development of the farrowing huts. Bonde et al. [8] did not mention fractures, and in general, very little is known about the causes of healed fractures found at slaughter. Healed rib fracture and old fracture usually lead to local condemnation [6].

#### Lesions with equal risk

Fresh fracture, chronic pneumonia, chronic pleuritis and abscess in the head or ear were recorded equally frequent in pigs from the two types of production systems. Fresh fractures are probably caused by trauma during delivery and transport to the abattoir. It results in local condemnation [6].

The equal risk of chronic pneumonia and chronic pleuritis probably reflects that the indoor-raising of organic/free-range pigs after weaning (Table 1) allows transmission of respiratory agents on a level similar to that in conventional production systems. This is in accordance with Heldmer et al. [9] who observed an increase in the prevalence of enzootic pneumonia in organic pigs over a period of 9 years and concluded that it was probably caused by a change from widespread outdoor raising to primarily indoor raising of organic finishing pigs during that period. The lesions recorded as chronic pneumonia are primarily complicated pneumonias, sometimes with abscessation. Uncomplicated enzootic pneumonia is generally not recorded. The primary pathogen behind pleuritis in Danish finishing pigs is *Actinobacillus pleuropneumoniae* (*App*). However, several other infectious agents also contribute, e.g. *M. hyopneumoniae* and *Pasteurella multocida*[28]. Since the majority of organic/free-range sow herds have an unknown status with regard to infection with *App* and *M. hyopneumoniae*, it is not known whether the proportion of pigs coming from infected sow herds are different from that in conventional herds (M.G. Christiansen, economist, Danish Pig Research Centre, personal communication June 2014). According to Bonde et al. [8] the prevalence of respiratory lesions was significantly lower in the 16 organic herds in comparison with the 52 conventional herds included in that study (11.6% versus 27.9%). The difference between Bonde et al.’s study and the present findings might be explained by sample size bias in the first study. In contrast, the present study included not just a limited sample of organic and free-range pigs but almost the entire Danish production of these pigs over a period of 1 year.

#### Lesions with lower risk in organic/free-range pigs

The prevalence of abscess in leg or toe (OR = 0.7) and scar and hock contusion (OR = 0.4) was lower in the organic/free-range pigs compared with the conventional pigs, probably because of the kind of floor surfaces and the use of bedding in the organic/free-range production (Table 1). A beneficial effect of solid floors and of the use of straw on the prevalence of bursitis on the hocks has been established [29]. In the same study, a significant association between the presence of bursitis and the presence of foot lesions was found. In the study by Bonde et al., abscesses were included either in the group “skin lesions or abscesses” or in “legs”, and it was not possible to determine into which of the two groups hock lesion belonged [8].

The prevalence of hernia (OR = 0.7) was lower in the organic/free-range pigs compared with the conventional pigs. No data were available allowing an explanation of this finding. Umbilical infection is probably a predisposing factor for some types of hernia [30], and perhaps the risk of umbilical infection is lower when pigs are born in farrowing huts. The mechanism behind this could be differences in the microflora in the farrowing huts compared with that of conventional farrowing pens, in part because the sows do not defecate in the huts. However, this is pure speculation. The finding was supported by Bonde et al. [8] who found a significantly lower prevalence of so-called intestinal lesions, typically consisting of hernia and peritonitis, among organic/free-range finishing pigs compared with conventional pigs (0.8% versus 1.4%).

### Limitations of study, further work and implications for stakeholders

This is the first time that a comprehensive set of data from meat inspection is being published, enabling a comparison of the health of finishing pigs from organic/free-range production and conventional production at the time of slaughter. However, the data structure only allowed a calculation of the over-all prevalence of each lesion recorded within each type of production system (organic/free-range versus conventional) and a subsequent comparison of these prevalences between production systems. Hence, it was not possible to distinguish between organic and free-range. This restriction was imposed to ensure the privacy of the farmers. Unfortunately, it also limited the statistical analysis and made it impossible to identify to which extent the variation observed in the prevalence of the individual lesions was caused by the individual herd. In other words: the identified differences might be ascribed to a limited number of the organic/free-range herds that have problems. On the other hand, this abattoir slaughters the main part of Danish, organic/free-range pigs as well as more than one million conventionally raised finishing pigs. Hence, the results obtained are not representing a sample of pigs taken from a limited number of organic or free-range herds.

The data structure also limits the investigation of other risk factors such as herd size and gender. Moreover, it was impossible to separate the effect of type of production system from the effect of tail-docking, because tail-docking is prohibited in organic/free-range production (unless exemption has been obtained) and widely practiced in conventional production.

Each individual pig might have suffered from several lesions at the time of slaughter. It would be of interest to follow up upon these data with a deeper analysis of the lesions indicated in Table 3 to determine to which extent the recordings revealed the presence of several lesions in the same animals. This would enable an identification of unique disease complexes (group of lesions that occur concurrently) for organic/free-range and conventional production, respectively, as suggested above. Such an investigation would also show ways of solving the challenges to each type of production system.

Some lesions are not observed at routine meat inspection because it requires the opening of an organ. One example is gastric ulcer. To investigate the prevalence of gastric ulcer, an extended meat inspection is required. In Denmark, this is ordered by the veterinary practitioner. Extended meat inspection is most typically done in the case of suspicion of gastric ulcers or pneumonia, and hence, it is used to monitor the health status. This type of investigation is paid for by the farmer. Similarly, cases of enteritis are not recorded routinely during meat inspection unless the lesions are visible from the outside surface of the intestines. Meat inspection will in general underestimate the prevalence of lesions in comparison with what is observed on the farm both because some lesions disappear before slaughter and some pigs die before slaughter.

It is known that the inter-observer variation of meat inspection varies between the lesions. According to Schleicher et al. [31] the variation is low for the following lesions: pericarditis, peritonitis, arthritis and milk spotted liver, whereas it is high for skin lesions and hepatitis. It might be argued that the more severe consequences that a lesion has for the destiny of the carcass (such as total condemnation), the more certain the recording of the lesion will be. The lesions described in Table 3 include lesions associated with both a high and a low variation. However, the source of variation was minimized by including only one abattoir plant.

There is a potential for improvement of both economy and welfare by preventing disease in all three types of production systems. For example, the pig farmers’ advisors– such as the veterinary practitioner and the pig production consultant – should be encouraged to make use of the data from meat inspection as also suggested by Sanchez-Vazquez et al. [32]. This might increase the attention on prevention of disease and injuries. For the organic and free-range producers this could also help them obtain the premium paid for these specialty products for an increased number of pigs and hereby improve the economy of the producer.

For conventional production it would be valuable to identify feasible ways of reducing injuries caused by the floors. The ban on fully slatted floors in Danish herds from 2015 might contribute to such reduction. It could also be valuable to identify methods of allocating bedding and to focus on how the slurry-handling systems can be further improved to enable use of straw or other types of bedding.

For the organic/free-range production, it would be relevant to evaluate whether treatment of e.g. tail lesions is undertaken when needed, and if recommendations regarding when to treat with antimicrobials could be set up and communicated to the producers to avoid under-treatment. As stated above, the current incentive structure for organic production – which implies that a pig is no longer considered organic if it has gone through two courses of treatments with antimicrobials – might have its drawbacks. Considering the rules for withdrawal time after antimicrobial treatment, there is minimal risk of antimicrobial residues in the meat of finishing pigs because the time of slaughter is known [33]. It could be debated whether it would be better to apply rules based on a quantification of the consumption of antimicrobials on herd level, e.g. in the form of a treatment index which is already developed in Denmark [34]. The individual pig could be sick or injured twice just by coincidence. To prevent wet resting areas and a humid pen environment, it would also be of interest to identify how proper drainage of urine and feces can be maintained when solid floors are in use. Moreover, a more widespread use of proper all-in all-out management and good cleaning and disinfection would probably be beneficial to the health and welfare of the pigs. However, on many farms this would probably require a change of the layout of the buildings allowing a better segregation of batches in individual rooms compared with what is seen today and preventing direct contact between different batches in the outdoor area.

The organic and free-range production is set up to favor the natural behavior of pigs (piglets born outdoors, more space, less confinement, possibility of rooting behavior etc.). However, fulfilment of behavioral needs does not necessarily result in good animal welfare if the animals suffer from poor health and vice versa. In the conventional production there is less focus on the behavioral needs of the animals and more focus on avoiding specific diseases and injuries by controlling the pen environment and preventing disease transmission. Hence there is a widespread use of tail-docking, sow crates, and slatted floors, and hygienic measures are applied routinely. This paper shows that there are challenges to both types of pig production systems. It makes limited sense to conclude whether one system is better than the other because they each have their advantages and disadvantages. Most importantly, focus should be on what is actually happening in a herd – reflected by objective, animal-based parameters such as meat inspection recordings – instead of attempting to evaluate the level of animal welfare in the herd, just on the basis of the requirements to the specific production system.

## Conclusion

The health at the time of slaughter was evaluated by use of meat inspection data from one abattoir covering 201,160 organic/free-range finishing pigs and 1,173,213 conventionally raised finishing pigs. The analysis showed statistically significant differences in the prevalence of the various lesions – measured as recordings by the meat inspectors – between the two types of pig production systems. Compared with conventional pigs, organic/free-range pigs had a higher prevalence of 13 lesion types. These lesions included, among others, fractures, tail lesions, and osteomyelitis. Four lesion types were equally common in the two production systems; among these were chronic pneumonia and chronic pleuritis. Finally, four lesions occurred less frequently in organic/free-range finishers compared with conventional finishers. These included abscesses in leg/toe, hernia and scar/hock lesion.

Apparently, the production systems each have their challenges which should be dealt with in collaboration between the farmer and his advisors.

## Methods

Data were obtained from the Danish slaughterhouse database. Meat inspection recordings from 1 year – from October 1, 2012, to September 26, 2013 - were included in the analysis. These covered 201,160 organic/free-range finishing pigs and 1,173,213 conventionally raised finishing pigs. The data were divided into organic/free-range and conventional pigs, respectively. These pigs originated from 54 organic herds, 117 free-range herds, and 789 conventional herds. The sows from all three types of production systems are slaughtered as conventional sows on separate plants, and sows were not included in the dataset. The number of times a given type of lesion was recorded as well as the total number of pigs inspected was given. Recordings that did not reflect pathological lesions were excluded, e.g. recordings due to lack of slap mark, colored skin, lack of ear tag, slaughter error, and contaminations.

All types of lesions occurring with a prevalence above 0.1% among the organic/free-range or conventionally raised pigs were included in the analyses. This corresponded to 99.4% of the recordings among organic/free-range pigs and 99.0% of the recordings among the conventional pigs. However, the lesion pyemia was included although the prevalence was lower than 0.1%, because acute pyemia results in total condemnation of the carcass. The following were among the lesions not included: nephritis, chronic gastritis/enteritis and neoplasia. All three conditions are less common in finishing pigs than in sows, e.g. a total of 56 cases of neoplasia (0.03%) were found among the organic/free-range pigs and 125 (0.01%) among the conventionally raised pigs.

First, the total prevalence of all lesions recorded was calculated for organic/free-range and conventional pigs, respectively. Here, it was ignored that one pig could be observed having more than one lesion type. Hence, it reflects the intensity of recordings in each of the two types of production system. Subsequently, the same calculation was done for the total of all lesions minus chronic pleuritis. A chi-square test comparing the prevalences for organic/free-range pigs and conventional pigs, respectively, was done.

Next, the prevalence of the individual lesion types was compared between the two groups (organic/free-range versus conventional) by use of a logistic regression model. The response was the number of pigs (y) found with a given lesion during the study period divided by the number of pigs slaughtered (x) in each group (organic/free-range versus conventional). This model was chosen because of the data structure, which listed y and x for each lesion type. The software program SAS version 9.3 was used.

Statistical analyses conducted on large datasets often reveal statistical significance although the biological difference seems to be unimportant. To counteract this, the P-value for significance was lowered from the customary 0.05 to P = 0.001, and only biological associations reflecting Odds Ratios (OR) above 1.2 or below 0.8 were considered of significance.

## Authors’ information

All three authors are trained as veterinarians and affiliated with the Danish Agriculture & Food Council. LA holds a Ph.D. in epidemiology and she is a diplomate of European College of Porcine Health Management as well as the European College of Veterinary Public Health. Her current position is Chief Scientist with responsibility for risk assessment and epidemiology. She is also adjunct professor at University of Copenhagen. JVP is a Senior Consultant and has for many years been involved in all issues related to transport and slaughter of pigs in Denmark. MEB has a M.Sc. degree in applied animal behavior and animal welfare. She manages applied research projects concerning production diseases and welfare in pigs.
